# 

**Published:** 1874-09

**Authors:** 


*To Our Subscribers.*--Our thanks are especially due those of our subscribers who have remitted their dues since the issue of the August number, and thus aided us in carrying out our contract with our publishers. Those who have not done us this favor, have put us to very great inconvenience as well as cost, insomuch as we are their surities for each number sent them. Will they not send their supscriptions immediately after receiving this number, and thereby relieve us during the "hard times?" Don't fail, friends.

☞ Write your Names, Post Office, County and State *plainly*. Be sure to say what Post Office you wish the RECORD sent, and always date your letters. We receive many well written letters, but doubt even the authors being able to decipher their signatures.

☞ Address all Communications, to POWELL & GOLDSMITH, none other.

☞ List of Cash Payments will appear in the next issue.

☞ Send Money by Check, Postal Order or by Registered Letters. We are willing to endorse for the honesty of our subscribers, but cannot be responsible for the irregularity of the mails.

*Medical Colleges.*

Our endorsement is fully and cordially given to the medical colleges announced in our advertising pages. The University of Pennsylvania and Bellvue Hospital Medical College are institutions so generally and thoroughly known to the profession, that the bare mention of their respective names is enough. Friends, practitioners and students, do you wish to honor your profession, and attain to the highest medical standing in this country? Then patronize institutions as have the professional and financial facilities—the men of high medical attainments, learned in their special branches and of national reputation, and having the largest clinical advantages from hospitals for the study of diseases. Let them remember that the light of a new day is breaking over the land, when half-made doctors will sink into obscurity and the educated men will reap the honors and rewards of the profession. If students desire to stand high, they must prepare themselves from the start and forecast the future with jealous scrutiny. P.

Dr. D. E. S.—" It is impossible to keep up a medical society in our town. A large majority of our physicians are too poor to sacrifice anything to sustain the right when it comes in conflict with their interest. We sometimes talk ethics; but seldom observe its requirements. Every man is for himself, consults with every class of doctors, and charges about what he thinks he can get. . . . We are a free set, with no money, and with little hope of better days, and especially so long as the colored people believe that the Civil Rights bill is intended to purify them and make them superior to the present white race—a people to be fed after the passage of the bill by the ravens, or so long as professors continue to graduate men who no kind of a bill or process could improve. . . . I hope you will excuse my neglect to submit sooner my dues to the Record. I could not do without it, and will be more punctual in the future."

We are sorry to hear even the truth, when the truth bears testimony against the professional irregularities of our brethren, the medical men of the land. We trust the demoralization has not swept away the honor of the profession. We feel that it has not. We are assured, in our esteem and high appreciation of our noble profession, that there are many, very many, true and high-toned professional gentlemen who have not bowed the knee to Baal. We hope they, in concert with all true men of all professions, will seek to elevate the standard of *morality*, and concentrate the good and true into one mighty lens, to fuse and utterly burn corruption from the land.

As to medical colleges, we, with our friends, deplore and lament the sad spectacle. In some sections of our country, they have sprung, fungus-like, into existence, and clamor for patronage.— They resort to every pretext, and hold out inducements for patronage to students in the most indefensible and reprehensible manner, violating every principle of ethics and morals. So far as we are concerned, we can simply look on, and, like Jeramiah, weep

### ASSOCIATE EDITORS:

GEORGIA.

James A. Long, M.D.

W. L. M. Harris, M.D.

H. L Battle, M.D.

W. C. Musgrove, M D.

L. B. Bouchelle, M.D.

Thomas Burdell, M.D.

E. H. W. Hunter, M.D.

V. S Hitt, M.D.

J. E. Pope, M.D.

A. H. Brantley, M.D.

J. J. Knott, M.D.

J. C. Wisdom, M.D.

W. W. Leake, M.D.

S. G. WHITE, M.D.

W. H. HALL, M.D.

G. D. CASE, M.D.

KENTUCKY.

W. S. Taylor, M.D.

B. W. Stone, M.D.

Charles Kearns, M.D.

TEXAS.

S. M. Welch, M.D.

T. J. Heard, M.D.

W. A. Heard, M.D.

TENNESSEE.

J. D. Tucker, M.D.

ALABAMA.

J. C. Knox, M.D.

Geo. F. Taylor, M.D.

J. B. Barnett, M.D.

S. M. Hogan, M.D.

D. S. Hopping, M.D.

J. T. Searcy, M.D.

S. F. Hill, M.D.

NORTH CAROLINA.

J. B. Hughes, M.D.

S. S. Satchwell, M.D.

A. B. Pierce, M.D.

H. Kelly, M D.

Geo. A. Foote, M.D.

E. A. Anderson, M.D.

H. T. Bahnson, M.D.

Willis Alston, M.D.

Thos. F. Wood, M.D.

N. J. Pittman, M.D.

John W. Booth, M.D.

SOUTH CAROLINA.

E. T. McSwain, M.D.

G. M. Rivers, M.D.

OHIO.

J. Q. A. Hudson, M.D.

C. H. Tidd, M.D.

VIRGINIA.

John H. Claiborne, M.D.

F. Horner, Jr., M.D.

R. J. Preston, M.D.

J. E. Lockridge, M.D.

J. S. Wharton, M.D.

Wm. O. Owen, M.D.

Sam'l P. Ripley, M.D.

Benj. Blackford, M.D.

E. A. Drewry, M.D.

Rob B. Tunstall, M.D.

W. F. Barr, M.D.

L. B. Anderson, M.D.

J. M. Lazzell, M.D.

MISSISSIPPI.

C. B. Gallaway, M.D.

Edward Lea, M.D.

S. V. D. Hill, M.D.

E. T. Henry, M.D.

T. P. Lockwood, M.D.

Robert Kells, M.D.

ARKANSAS.

A. D. Hodge, M.D.

J. K. Hodge, M.D.

A. W. Hobson, M.D.

NEW JERSEY.

J. B. MATTISON, M.D.

### CORRESPONDING EDITORS:

Ely Van DeWarker, M.D., New York.

C. C. F. Gay, M.D., New York,

*Surgeon of Buffalo General Hospital.*

M. H. Henry, M.D., New York,

*Surgeon to N. Y. Dispensary, Dep't of Venerial and Skin Diseases.*

Benjamin Howard, M.D., New York.

James Tyson, M.D., Philadelphia, Pa.

W. W. Keen, M.D. Philadelphia.

Robert Robson, M D., Indiana.

A. Patton, M.D., Indiana.

John H. Weir, M.D., Illinois.

Thomas J Gallaher, M.D, Pennsylvania.

S. C. Busey, M.D., Washington, D. C.

Wm. R. Whitehead, M.D., Colorado Territory.

